# The levels of urine CTX-II, C2C, and PYD in children patients with Kashin-Beck disease in Qinghai Province of China

**DOI:** 10.1186/s13018-018-1057-x

**Published:** 2019-01-11

**Authors:** Wanying Wang, Lihua Wang, Qing Deng, Yun Cai, Xianhao Wu, Liyan Sun

**Affiliations:** 1 Center for Endemic Disease Control, Chinese Center for Disease Control and Prevention, Harbin Medical University, 157 Baojian Road, Harbin, 150081 China; 2 Key Laboratory of Etiology and Epidemiology, Education Bureau of Heilongjiang Province & Ministry of Health (23618504), Harbin, 150081 China

**Keywords:** Kashin-Beck disease, Early diagnosis, Urine, CTX-II, C2C, PYD

## Abstract

**Background:**

Kashin-Beck disease (KBD) is an endemic and chronic osteoarthropathy. At present, the diagnosis of KBD mainly depends on the X-ray examination and which could not reflect early damage of cartilage sensitively. So, the aim of this study was to find effective and sensitive biomarkers for early diagnosis of pediatric KBD.

**Methods:**

A total of 122 children aged 7–15 years old from 3 villages of Qinghai Province were eligible for the study. Thirty-one, 41, and 50 children were assigned in case, internal, and external control groups, respectively. The levels of CTX-II, C2C, and PYD in urine were measured by using ELISA and compared statistically. In addition, the receiver operating characteristic curve (ROC) analysis was used to assess the performance of diagnostic biomarkers.

**Results:**

There were significant differences in levels of CTX-II, C2C, and PYD in urine of subjects among three groups. The levels of CTX-II and PYD in the case group were significantly higher than those in external and internal control groups. On the contrary, the level of C2C in the case group was lower than that in the external control group. Compared to the external control group, the area under the curve (AUC) of CTX-II, C2C, and PYD were 0.857, 0.837, and 0.79, and the AUC of CTX-II significantly higher than that of PYD. Compared to the internal control group, the AUC of CTX-II, C2C, and PYD were 0.911, 0.875, and 0.839, and there were no significant differences in the AUC among three indicators.

**Conclusion:**

Both CTX-II and PYD in urine could be used as biomarkers for early diagnosis of pediatric KBD, and the prediction accuracy of CTX-II was relatively superior.

## Background

Kashin-Beck disease (KBD) is a special, endemic, chronic, and deformative osteoarthropathy [1]. It is clinically characterized by arthralgia, joint enlargement, joint deformation, muscle atrophy, even short fingers (toes), and short limbs, and the most serious is pygmyism and malformation [2]. KBD are similar to osteoarthritis (OA) in certain clinical manifestation and pathological cartilage degeneration [3]. However, it differs from common OA particularly in the onset age of the disease: KBD occurs predominantly at 5–15 years of age [4].

KBD has a high prevalence in the broad diagonal belt from northern-east to southern-west in China [4]. In the past few decades, the Chinese government has launched a massive effort and series of measures to prevent and control KBD [5, 6], and the incidence of KBD has been declined markedly. But according to the national monitoring data, new patients continue to occur in some western regions of China, particularly in the Qinghai Province, Inner Mongolia, and Tibet Autonomous Regions.

As an endemic disease, KBD is relatively easy to prevent and difficult to cure. In this situation, the prevention and early diagnosis of disease is particularly important. At present, the diagnosis of KBD usually depended on the X-ray examination (the present golden standard to diagnose KBD) [7]. However, the X-ray examination is not sensitive to the early pathological changes. In other words, it is possible that the cartilage has been severely damaged, but the X-ray examination still appears normal [8, 9]. So, some other ways to evaluate the early cartilage changes of KBD need to be explored. Previous studies showed that comparing with healthy ones, there was a significant difference in various indicators in the patients with KBD, but so far, it was not found that a single index was specific to the early diagnosis of the disease [10].

Biological fluids, such as urine and blood, contain many indicators, which can provide a large amount of biological information about body status [11]. Compared to blood sample, urine sample collecting is not traumatic to human body and easier to do. In many OA studies, C-telopeptide of type II collagen (CTX-II), type II collagen cleavage neoepitope (C2C), and pyridinoline (PYD) in urine are often used to assess the extent of cartilage damage [12–14]. All of them are relative useful indicators that could provide some information about metabolic changes and turning over of joint cartilage. As far as we know, few studies have measured the change of CTX-II, C2C, and PYD in urine of children and adolescent patients with KBD simultaneously.

In order to find more effective and sensitive biomarkers in urine for early diagnosis of pediatric and adolescent KBD, the levels of CTX-II, C2C, and PYD in urine were measured and compared among healthy children and pediatric KBD patients in present study. Furthermore, the prediction accuracies of these three indicators as diagnostic markers were assessed.

## Materials and methods

### The selection of study sites and subjects

According to the national monitoring data of KBD, some relatively active KBD endemic areas and non-KBD endemic areas including Tangnaihai Village and Qushan Village of Xinghai County, Qinghai Province, and Gandu Village of Hualong County, Qinghai Province, were selected as our study sites.

Regular health and radiologic examinations were performed for all children aged 7–15 years old who were living and studying in the boarding school of our study sites. Both the radiologic examinations and KBD diagnosis were performed according to the Chinese radiologic and clinical diagnostic criteria for KBD [15]. After the examinations, the eligible individuals were selected. Children with other joint diseases (such as joint inflammation, metabolic bone diseases, neoplasia, osteoporosis, or osteomalacia), joint damage and injury, progressive joint surgery within a year, taking arthritis drugs nearly a month, suffering from chronic systemic diseases and acute inflammation, and those who declined to participate in this study were excluded. In addition, the living standard and habit of control groups were similar to case groups as far as possible in order to exclude the influence from other special factors on research results.

Ultimately, 122 children were selected in this study cohort: 31 KBD children with metaphyseal changes of fingers entered in the case group, 41 healthy children from KBD endemic areas were assigned in the internal control group, and 50 healthy children from non-KBD endemic areas entered in the external control group.

### Sampling, processing, and detection of urine samples

The morning urine samples (not less than 5 mL/person) of children were collected in 15-mL sterile tubes. The urine samples were left at room temperature for 30 min and then were centrifuged at 3000 rpm/min for 10 min to separate the supernatant. The supernatant was dispensed into 200 μL micro centrifuge tubes that were stored at − 80 °C until the assay.

The levels of CTX-II, C2C, and PYD of subjects in different groups were quantified by enzyme-linked immune sorbent assay (ELISA) in accordance with the manufacturer’s instructions (Wuhan USCN Business Co., Ltd., Wuhan, China). The concentrations of the three urine indicators were normalized by urinary creatinine levels. The intra-assay and inter-assay coefficient variations of all indicators were less than 15%, respectively.

### Statistical analysis

The data was analyzed by SPSS 20.0 software. Kolmogorov-Smirnov test was used to check if the data conforms to normal distribution (*α* = 0.05). Data obey the normal distribution was described with mean and standard deviation, and the non-normal data was described with the median and interquartile range. After the homogeneity test of variance (Levene’s test), the significant difference analysis of data fitted the normal distribution was analyzed by the one-way analysis of variance (ANOVA), and the non-normal data was analyzed by Kruskal-Wallis H test. LSD test and Nemenyi test were used for the comparison of any two groups, respectively. The receiver operating characteristic curve (ROC) analysis was used to determine the performance of diagnostic biomarkers. From each ROC, the area under the curve (AUC) represents the prediction accuracy of indicators. All the tests were two-tailed, and *P* < 0.05 was considered statistically significant.

## Results

### The basic characteristics of subjects in each group

Basic characteristics include subject’s sex, age, and BMI in different groups are described in Table 1. Sex and BMI of subjects were balanced and comparable among three groups (*P* > 0.05). And there was a significant difference in age of subjects among three groups (*P* < 0.001).

**Table 1:** The basic characteristics of subjects in different groups (mean ± SD, median, interquartile range)

	*N*	Sex (male/female)	Age (years)	BMI (kg/m^2^)
External control group	50	25/25	10.0 (8.0, 10.0)	16.01 ± 1.76
Internal control group	41	24/17	11.0 (10.0, 12.0)	16.68 ± 1.96
Case group	31	14/17	12.0 (10.0, 13.0)	16.15 ± 1.73
*P* value		0.709	0.000	0.249

### The levels of CTX-II, C2C, and PYD of subjects in different groups

**Fig. 1:**
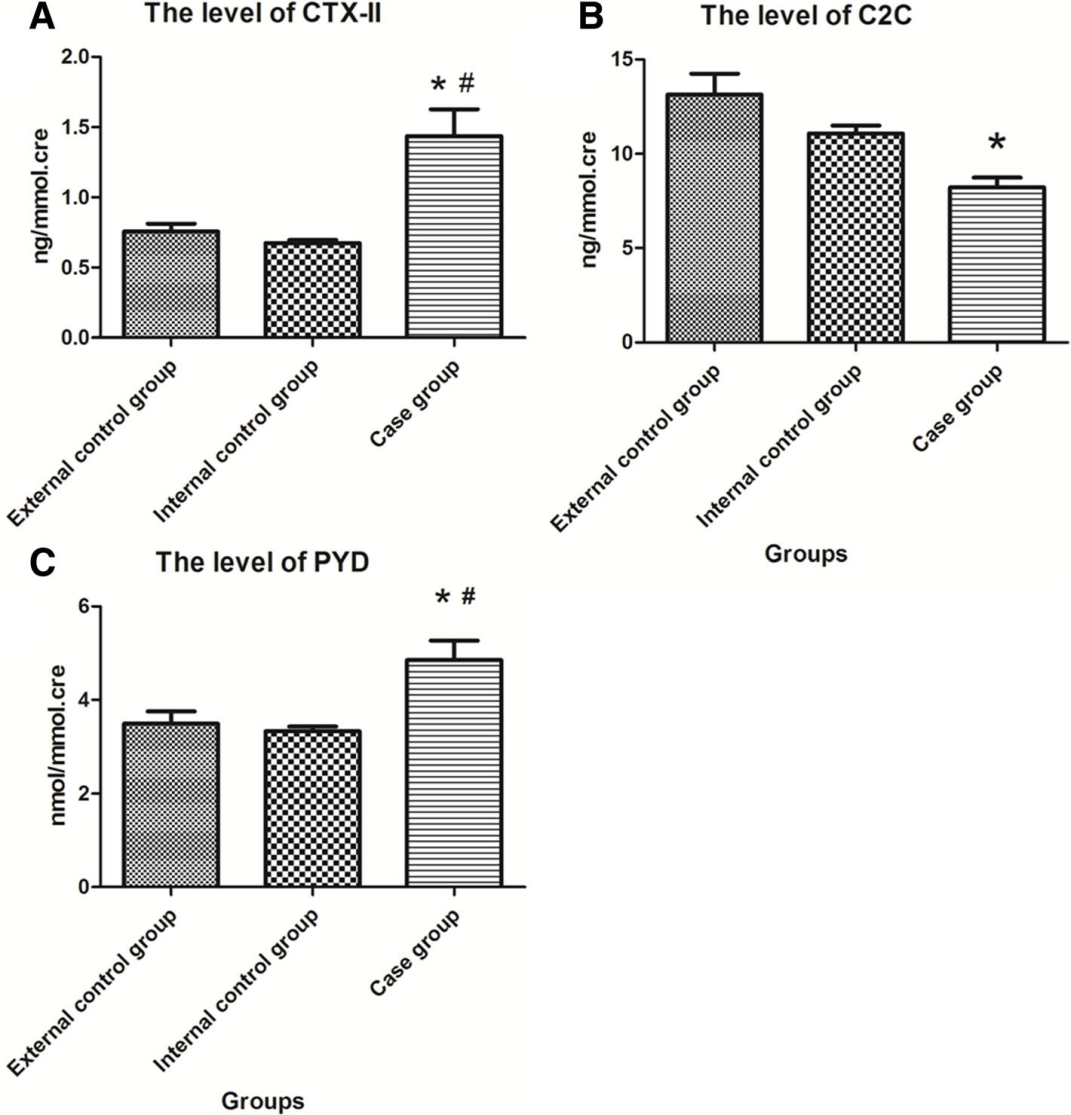
The levels of CTX-II, C2C, and PYD in urine of different groups. Part **a**, part **b,** and part **c** showed the level of CTX-II, C2C, and PYD in urine of three groups, respectively, and all data of three indicators conform to normal distribution. *Means *P* < 0.05 when compared to the external control group, ^#^means *P* < 0.05 when compared to the internal control group

The levels of CTX-II in the external control group, internal control group, and case group were 0.72 ± 0.22 ng/mmol.cre, 0.67 ± 0.09 ng/mmol.cre, and 1.30 ± 0.48 ng/mmol.cre, respectively. Significant differences were seen among all three groups (*F* = 21.48, *P* < 0.001), and the level of CTX-II in the case group was obviously higher than those both in the external and internal control groups (*P*_external control group vs case group_ = 0.007, *P*_internal control group vs case group_ = 0.004). However, there was no statistical difference in the level of CTX-II between the external control group and internal control group (*P* = 0.757), the data was present in Fig. 1a.

The levels of C2C in the external control group, internal control group, and case group were 13.13 ± 5.59 ng/mmol.cre, 11.09 ± 1.68 ng/mmol.cre, and 8.22 ± 1.82 ng/mmol.cre, respectively. There were significant differences in the level of C2C among all three groups (*F* = 5.95, *P* = 0.005). As opposed to the level of CTX-II, the level of C2C in the case group was clearly lower than that in the external group (*P* = 0.001), but there is no significant difference in the level of C2C between the case group and the internal control group (*P* = 0.071). Similar to CTX-II, there was no distinct difference in the level of C2C between the external control group and internal control group (*P* = 0.124), the detail was shown in Fig. 1b.

The levels of PYD in the external control group, internal control group, and case group were 3.49 ± 1.30 nmol/mmol.cre, 3.33 ± 0.4 nmol/mmol.cre, and 4.85 ± 1.45 nmol/mmol.cre, respectively. Significant differences were seen among all three groups (*F* = 7.12, *P* = 0.002). The level of PYD in the case group was significantly higher than those in the external and internal control groups (*P*_external control group vs case group_ = 0.035, *P*_internal control group vs case group =_ 0.012), but no evident difference was seen between the external control group and the internal control group (*P* = 0.915, Fig. 1c).

### Assessment of the performance of three indicators in diagnosis for KBD

**Fig. 2:**
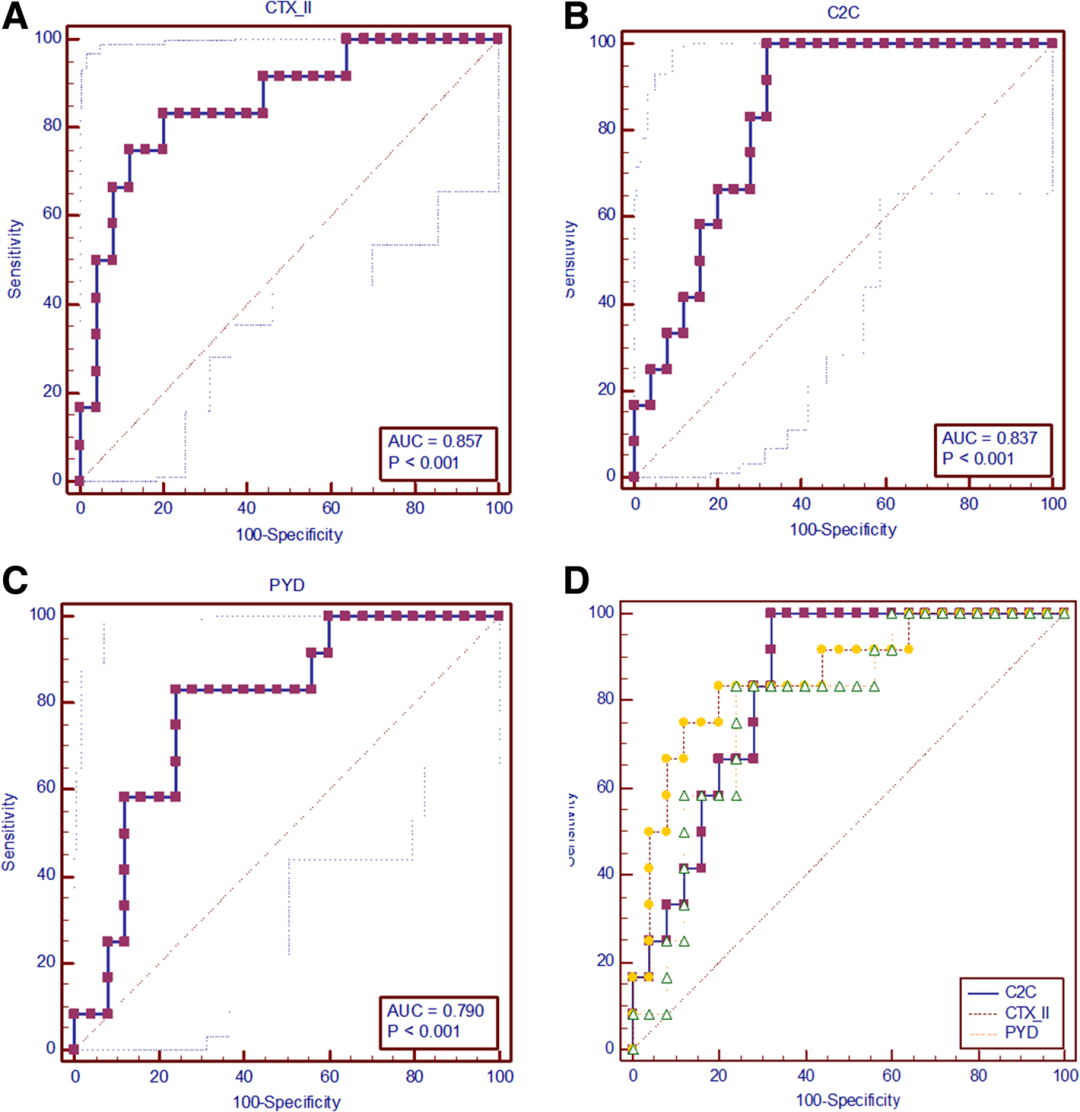
Prediction accuracies of three indicators for the early diagnosis of KBD compared to the external control group. Part **a**, part **b**, and part **c** showed the ROC curves and AUC of CTX-II, C2C, and PYD, respectively. Part **d** showed the comparison of ROC curves and AUC of CTX-II, C2C, and PYD

All three indicators showed good prediction accuracy for KBD compared with the external control group. Compared to the external control group, the AUC of CTX-II, C2C, and PYD were 0.857 (95% CI 0.702–0.95, Fig. 2a), 0.837 (95% CI 0.678–0.937, Fig. 2b), and 0.79 (95% CI 0.625–0.906, Fig. 2c), respectively. The AUC of CTX-II significantly higher than that of PYD (*P* = 0.041), but there were no significant differences in AUC between CTX-II and C2C (*P* = 0.803) or PYD and C2C (*P* = 0.584), the detail was shown in Fig. 2d.

**Fig. 3:**
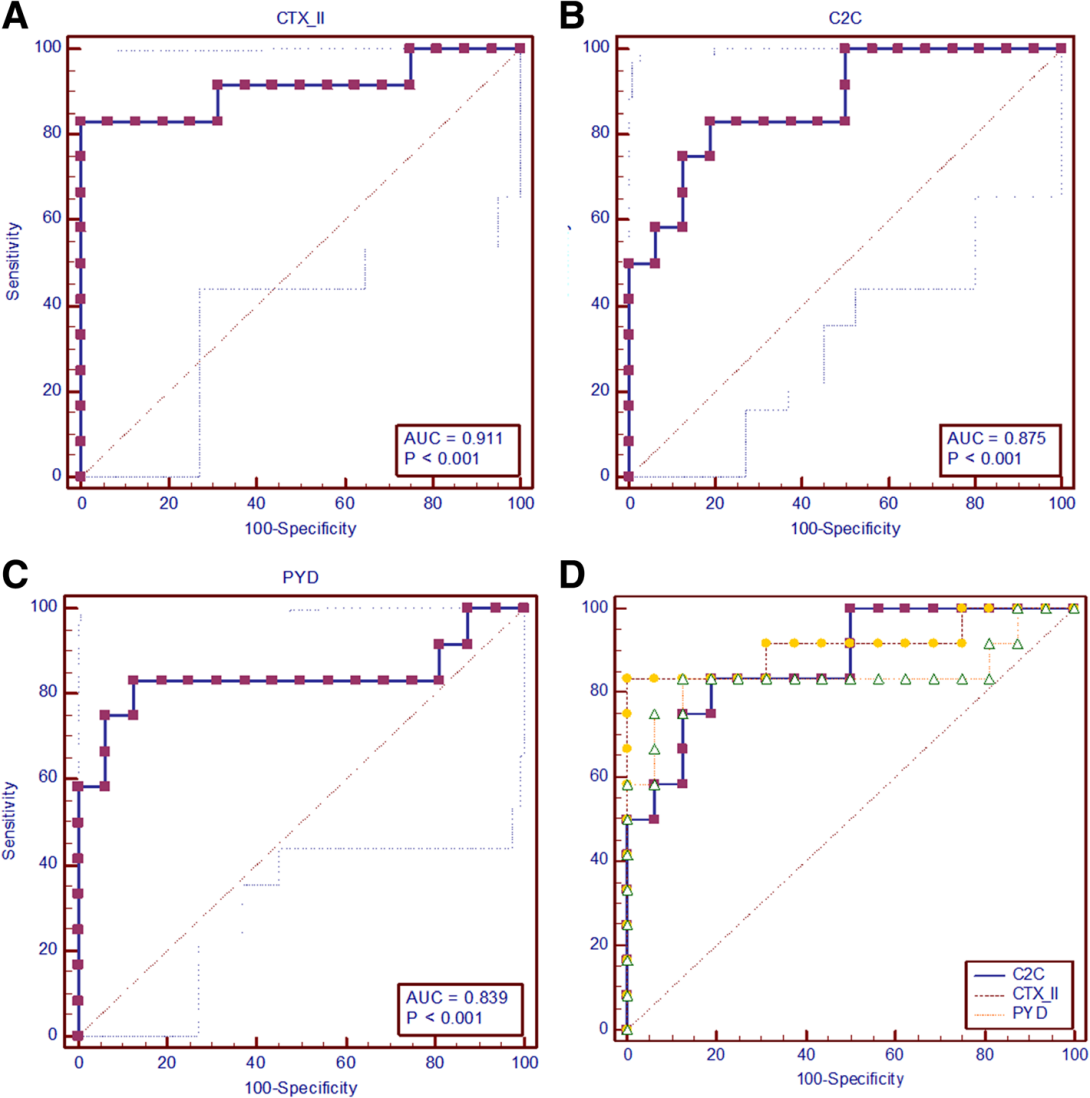
Prediction accuracies of three indicators for the early diagnosis of KBD compared to the internal control group. Part **a**, part **b**, and part **c** showed the ROC curves and AUC of CTX-II, C2C, and PYD, respectively. Part **d** showed the comparison of ROC curves and AUC of CTX-II, C2C, and PYD, and AUC of CTX-II and C2C were sharply higher than PYD (*P*_CTX-II vs PYD_ = 0.008, *P*_C2C vs PYD_ = 0.013)

Compared to the internal control group, the AUC of CTX-II, C2C, and PYD were 0.911 (95% CI 0.742–0.985, Fig. 3a), 0.875 (95% CI 0. 0.695–0.969, Fig. 3b), and 0.839 (95% CI 0.651–0.949, Fig. 3c), respectively. There were no significant differences in AUC among these three indicators (*P*_CTX-II vs C2C_ = 0.562, *P*_CTX-II vs PYD_ = 0.102, *P*_C2C vs PYD_ = 0.673, Fig. 3d).

## Discussion

Due to the incidence of KBD is decreasing year by year, the number of patients was small and the age of patients was relatively older, which results in the unbalance of age among groups. But gender and BMI, the major factors affecting the disease, were balanced and comparable. In addition, all children of this study were boarding students, and the diet was rationing by government, and the influence of different diet on the indicators was considered to be minor. Thus, the difference of age was considered had little influence on the test results.

The primary pathologic changes of KBD are chondrocytic degeneration, apoptosis, necrosis, and losses in the deep-zone of articular and growing plate cartilage, and with progress of disease, the catabolism of extracellular matrix (ECM) accelerated [16]. So, the early stage of KBD is manifested as osteochondrosis, and then, all changes develop continuously, ultimately progresses to OA [17].

Type II collagen is the most abundant constituent of ECM. CTX-II can be measured in urine providing a sensitive and specific measure of type II collagen breakdown [18]. Previous study showed that the level of CTX-II in urine might reflect the extent of type II collagen degradation in OA patients [14, 19]. Recent study of Zhao, et al. found that the level of CTX-II in urine of adult KBD patients clearly increased [20]. In our study, compared to external and internal control groups, the level of CTX-II in the case group elevated obviously. Although the patients were different, the result was consistent with Zhao’s study [20]. At the same time, whether compared with the external control group or the internal control group, the AUC of CTX-II were both highest, obviously higher than the AUC of C2C and PYD. Especially when compared to the external control group, the AUC, namely the prediction accuracy of CTX-II was significantly higher than that of PYD. Therefore, the above results jointly suggested that the level of CTX-II in urine should be a more powerful and sensitive marker for early diagnosis of KBD.

C2C is a degradation of 3/4 fragment of collagen II. Previous studies have shown that the level of C2C may reflect the extent of cartilage degradation and significantly increased both in serum and urine of OA patients [21–23]. However, the level of C2C in urine of children KBD patients has not been reported before. In this study, the level of C2C in case group decreased apparently, and this is contrary to previous studies. This might be because pediatric patients remain in the early stage of KBD (osteochondrosis) and do not progress to osteoarthritis. So, the role of C2C played in KBD needs further study. Similar to CTX-II, whether compared with the external control group or the internal control group, the AUC of C2C was greater than 0.8, lower than that of CTX-II, and higher than PYD, and this provided a valid clue for future KBD studies.

PYD derive from the breakdown of collagen. Due to its specific localization in the bones and renal excretion, the level of PYD in urine could help us better understand pathological changes in the bone and cartilage [24]. Previous studies found that the average level of PYD in urine of moderate adult KBD patients was higher than those of mild adult KBD patients and healthy control [10]. In our children patients, the level of PYD in the case group were significantly higher than those in external and internal control groups. When compared to external and internal control groups, the AUC of PYD were 0.79 and 0.839, and they were both greater than 0.7. These results showed that the level of PYD in urine also was a particularly valuable biomarker for early diagnosis of KBD. However, when compared to the external control group, the AUC of PYD distinctly lower than that of CTX-II. So, we thought that the PYD was less effective than CTX-II for early diagnosis of KBD.

However, this study has a number of limitations. First, the sample size of this study was comparatively small and only met the basic statistical requirements. The statistical efficiency could have been improved if the sample size had been appropriately increased. Secondly, all subjects were from Qinghai Province and there might be regional limitations. Therefore, we will continue to conduct further study in the future to verify the authenticity of this study.

## Conclusions

In summary, our study was unique because we firstly identified that CTX-II and PYD in urine could be used as biomarkers in early diagnosis of pediatric KBD, and the prediction accuracy of CTX-II was most powerful. The results also showed that C2C might be valuable to diagnose KBD early.

## Abbreviations

ANOVA: One-way analysis of variance
AUC: Area under the curve
C2C: Type II collagen cleavage neoepitope
CTX-II: C-telopeptide of type II collagen
ECM: Extracellular matrix
KBD: Kashin-Beck disease
OA: Osteoarthritis
PYD: Pyridinoline
ROC: Receiver operating characteristic curve
